# Targeting Notch Trafficking and Processing in Cancers

**DOI:** 10.3390/cells9102212

**Published:** 2020-09-29

**Authors:** Luca Pagliaro, Claudia Sorrentino, Giovanni Roti

**Affiliations:** Department of Medicine and Surgery, University of Parma, 43126 Parma, Italy; luca.pagliaro@unipr.it (L.P.); claudia.sorrentino@unipr.it (C.S.)

**Keywords:** NOTCH1, SERCA, T-cell acute lymphoblastic leukemia, thapsigargin, trafficking, CAD204520

## Abstract

The Notch family comprises a group of four ligand-dependent receptors that control evolutionarily conserved developmental and homeostatic processes and transmit signals to the microenvironment. NOTCH undergoes remodeling, maturation, and trafficking in a series of post-translational events, including glycosylation, ubiquitination, and endocytosis. The regulatory modifications occurring in the endoplasmic reticulum/Golgi precede the intramembrane γ-secretase proteolysis and the transfer of active NOTCH to the nucleus. Hence, NOTCH proteins coexist in different subcellular compartments and undergo continuous relocation. Various factors, including ion concentration, enzymatic activity, and co-regulatory elements control Notch trafficking. Interfering with these regulatory mechanisms represents an innovative therapeutic way to bar oncogenic Notch signaling. In this review, we briefly summarize the role of Notch signaling in cancer and describe the protein modifications required for NOTCH to relocate across different subcellular compartments. We focus on the functional relationship between these modifications and the corresponding therapeutic options, and our findings could support the development of trafficking modulators as a potential alternative to the well-known γ-secretase inhibitors.

## 1. Introduction

In 1914, John S. Dexter described a heritable “perfect notched” phenotype in beaded *Drosophila* [1]. Thomas Hunt Morgan originally reported that mutant “beaded” wings appeared in 1910 from fly stocks that had been exposed to radium rays early in their lives [2,3]. In 1917, he showed that the “notch” phenotype was caused by a heterozygous deletion of a gene located on the X chromosome that was subsequently named “*NOTCH*” after the notched shape [4]. Only decades later, Spyros Artavanis-Tsakonas [5] and Michael Young [6] cloned the NOTCH receptor in the same experimental model and provided an initial description of the structure and functions of NOTCH [6]. Since then, the contribution of the Notch signaling has been described in many physiological processes, from embryogenesis to adult tissue commitment and homeostasis [7]. In the same way, *NOTCH* aberrancies have been reported in several diseases, including inherited syndromes [8] and numerous types of cancer [9].

## 2. The NOTCH Structure

In mammals, the “Notch family” comprises a group of four transmembrane receptor proteins (NOTCH1-4) that interact with ligands that are expressed in contacting cells. The ligands comprise three delta-like (Dll1, Dll3, and Dll4) and two jagged (Jag1 and Jag2) type I transmembrane proteins [10]. NOTCH receptors share a modular structure, which is assembled into functional regions: an extracellular domain (N-ECD), an intracellular tail, and a connector between the two. Human NOTCH1 and NOTCH2 N-terminal domain N-ECD contains 36 homologous epidermal growth factor (EGF)-like tandem repeats and three Lin-12/Notch units (LNR), while that of NOTCH3 and NOTCH4 contains 34 and 29 repeats, respectively [11]. Adjacent to the LNR domain lies the juxtamembrane heterodimerization domain (HD), the linker between the N-ECD and NOTCH intracellular domain (N-ICD). N-ICD consists of a membrane proximal RBPJ-associated molecule (RAM) region, seven ankyrin-like repeats (ANK) [12,13], and a C-terminal transactivation domain (TAD) that harbors the nuclear localization sequences (NLS). The catalytic activity relies on presenilin (PSEN), which releases an N-ICD degradation domain containing the PEST sequence, a peptide sequence rich in proline, glutamate, serine, and threonine.

While the nuclear role of NOTCH has been thoroughly investigated [14], the cytoplasmic contribution of these proteins to physiological or tumor pathways is far from being completely understood. In this review, we focus on the mechanisms that control the Notch secretory pathway and the ways to exploit them for cancer therapeutics.

## 3. From Golgi to the Cell Membrane

**Figure 1:**
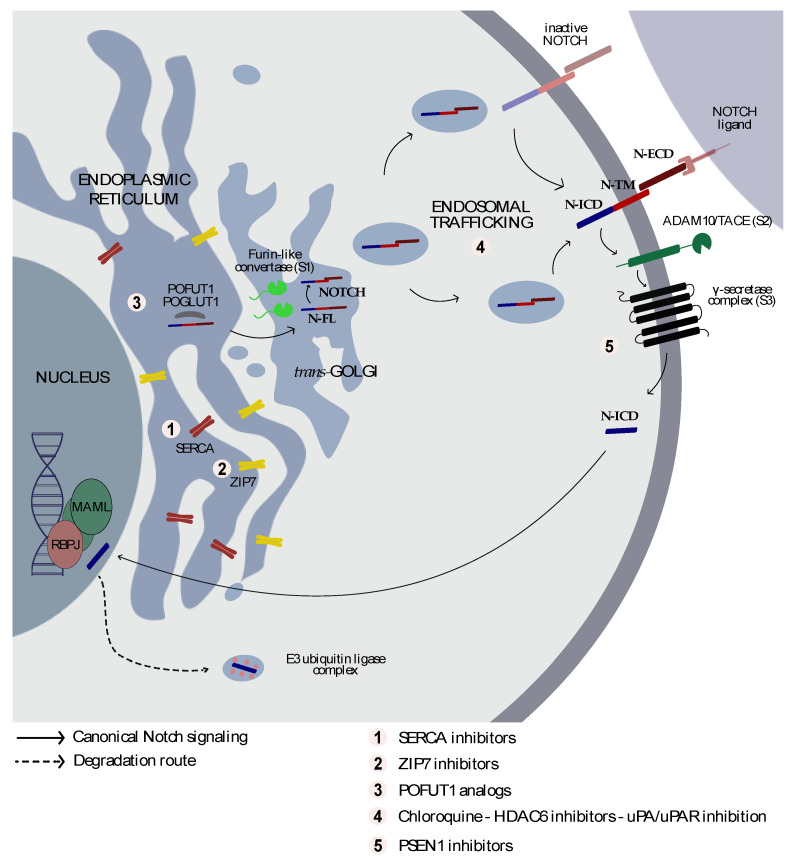
Schematic representation of Notch signaling and proteolytic processing. NOTCH receptors are cell surface receptor proteins. Interaction with mutual ligands activates two consecutive proteolytic cleavages at the extracellular site, one by a metalloprotease (ADAM10/TACE) (S2), followed by another by a γ-secretase (S3), resulting in the release of N-ICD. N-ICD is translocated into the nucleus where it interacts with a transcription-activating complex. In the presence of *NOTCH1* mutations, N-ICD is constitutively active, independent of ligand binding. The figure shows an overview of Notch trafficking routes and the corresponding targets for therapeutic intervention. N-FL: NOTCH full length; N-TM: NOTCH transmembrane; N-ICD: NOTCH intracellular domain.

The canonical Notch signaling pathway initiates in response to NOTCH ligands. The strength of receptor–ligand interactions and the fate of the nuclear signal are modulated by post-translational modifications occurring in the extracellular portion of NOTCH proteins [15] (Figure 1). The EGF-like repeats can be modified by the addition of an *O*-glucose or *O*-fucose transferred by the POGLUT1 (protein *O*-glucosyltransferase 1) [16,17,18] and POFUT1 (protein *O*-fucosyltransferase 1) [19,20], respectively. Interestingly, the combined addition of *O*-glucose or *O*-fucose stabilizes the EGF repeats in an additive manner and contributes to the cell surface expression of endogenous NOTCH1 in human embryonic kidney (HEK) 293T cells [21]. In mammalian NOTCH receptors, *O*-glucose glycans may be further modified by the addition of xylose residues at each of the EGF repeats [22]. Glucoside α3-xylosyltransferases (GXYLT1 and GXYLT2) and xyloside α3-xylosyltransferase (XXYLT1), for example, extend the *O*-glucose monosaccharides added by POGLUT1 [23]. Recent data has demonstrated that 70% or more of the NOTCH1 and NOTCH2 *O*-glucose glycoforms on 12 out of 17 EGF repeats are xylose-extended [24]. In mechanistic studies, the authors demonstrated that xylosyl extension by GXYLT1 and GXYLT2** is not necessary for the trafficking of endogenous NOTCH1 and NOTCH2 in HEK293T cells, while it is required for a proper protein trafficking in cells overexpressing NOTCH1 and NOTCH2 [24].

The accumulation of *O*-fucose primes NOTCH receptors for the further formation of glycosylated NOTCH intermediates by members of the Fringe family (Radical (R), Manic (M), Lunatic (L)) in the trans-Golgi compartment. Fringe proteins add *N*-acetylglucosamine (GlcNAc) sugars to the *O*-fucose moiety. The pattern of fucose modifications modulates the relative response of NOTCH receptors to ligands of the Delta versus Jagged/Serrate classes. Fringe proteins, for example, potentiate interactions with Dll1 and reduce the responsiveness to Jag1 [25,26,27]. Takeuchi and colleagues also identified two novel *O*-glucosyltransferases, POGLUT2 and POGLUT3, which are responsible for the transfer of UDP-glucose on the 11th EGF repeat on serine 435, a distinct site from that of POGLUT1 [17]. Mutations occurring in Ser435 affect the NOTCH1 cell-surface presentation and, consequently, its activation upon interaction with Dll1 [28]. 

During these maturation steps, full-length NOTCH (N-FL) proteins are subsequently cleaved within the so-called negative regulatory region (NRR) by a furin-like convertase at site “S1” in the trans-Golgi network. Proteolysis generates heteromeric single-pass transmembrane proteins of N-ECD that are non-covalently bound to the transmembrane NOTCH (N-TM) [29] and unstable in the presence of increasing concentrations of ethylenediaminetetraacetic acid (EDTA). EDTA mimics NOTCH activation [30], suggesting that disruption of Ca^2+^ homeostasis may alter the NOTCH processing [30]. As expected, furin knockout decreases NOTCH cleavage [31], as mutations occurring in the furin recognition sites [31]. It has also been suggested that furin processing cooperates with the Nodal pathway to enhance the post-translational maturation of NOTCH receptors. Nodal is a secretory protein that belongs to the transforming growth factor-beta (TGF-β) family and plays an essential role in vertebrate development [32]. Nodal binds type I and type II serine/threonine kinase receptors using Cripto-1 as a co-receptor [33] to mediate the final translocation of SMAD4 (SMAD family member 4, Mothers against decapentaplegic homolog 4) into the nucleus and the transactivation of genes involved in proliferation and differentiation processes [33]. From a two-hybrid screen to isolate Cripto-1 binding partners, Watanabe and colleagues initially identified NOTCH3 among other potential targets. Confirmatory studies showed that all NOTCH isoforms bind to Cripto-1 and that in the human embryonic carcinoma cell line NTERA2/D1 cells, Cripto-1 knockdown significantly suppresses the induction of the Notch target genes hairy and enhancer of split (*HES-1*) and hairy and enhancer of split-related with YRPW motif (*HEY-1* and *-2*) after NOTCH ligand stimulation. In fact, N1-FL accumulates in Cripto-1-deficient cells, suggesting that the latter is required for proteolytic processing [34]. Collectively, these data suggest that these modifications are regulated at multiple levels: ion fluxes, enzymes, and co-regulators, providing several opportunities for therapeutic intervention (Figure 1).

## 4. From the Cell Membrane to the Nucleus

Once preprocessed in the ER/Golgi, NOTCH receptors migrate to the cell membrane and receive an activation (trans) or inhibitory (cis) signal [35] after ligand interaction. This binding induces a conformational change to clip off N-ECD [36,37,38]. This proteolytic cleavage (S2) is mediated by a zinc-dependent disintegrin and metalloprotease (ADAM10 or 17) and occurs within the NOTCH juxtamembrane extracellular domain, proximal to the N-TM, between residues Ala1710 and Val1711 of NOTCH1 [36,37]. Different roles of the metalloproteases have been reported. ADAM10 cleaves NOTCH upon a canonical activation, while ADAM17/TACE releases the N-TM of mutant peptides [39,40]. The short-lived S2 fragments are the substrate of the γ-secretase complex [41,42] (S3), a membrane-embedded protease complex comprising the enzyme PSEN1 or PSEN2 with presenilin enhancer-2 (PEN-2), anterior pharynx defective-1 (APH-1), and nicastrin (NCSTN) [43,44]. The active γ-secretase consists of APH-1 and PEN-2 directly linked to PSEN and NCSTN [45]. The catalytic activity relies on PSEN, which releases an N-ICD from, such as Gly1743 and Val1744 of N1-TM [41]. The RAM and ANK N-ICD motifs bind the DNA factor suppressor of hairless [Lag-1 or CSL (mammalian RBPJ)] and recruit the N-terminal helices of mastermind-like (MAML) family co-activator to generate a transcriptional complex (N-TC) [46]. In succession, MAML recruits the histone acetyl-transferases (p300/CBP) to transcribe, among other genes, *HES*, *HEY*, and *MYC* [47,48]. 

## 5. Endocytosis and Trafficking

**Figure 2:**
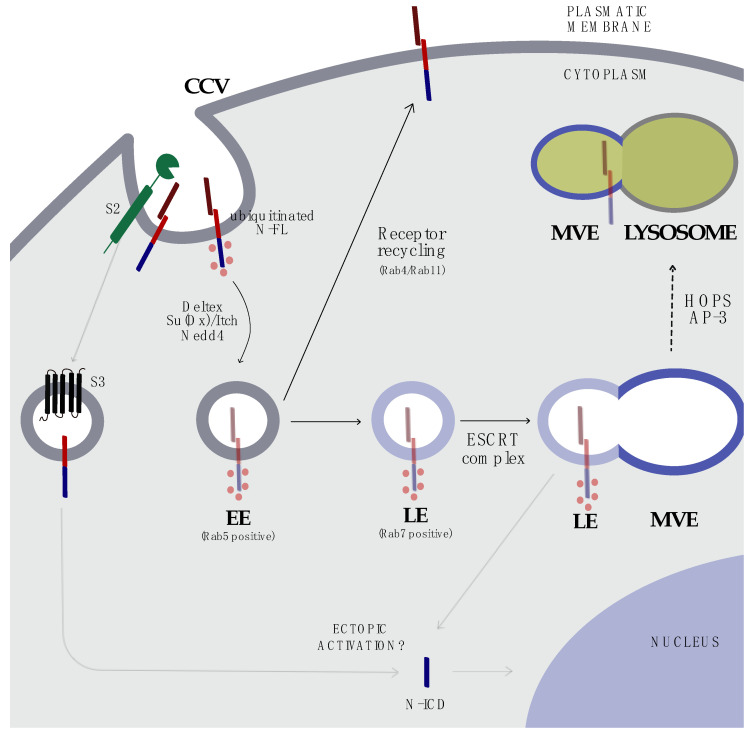
Overview of NOTCH endocytosis and vesicular trafficking. NOTCH proteins are monoubiquitinated by Deltex, Su(Dx)/Itch, Nedd4, and E3 ligase and endocytosed in clathrin-coated vesicles (CCV), resulting in Rab5-positive early endosomes (EEs). EEs might follow two different routes: (i) recycling back to the membrane through a Rab4/Rab11-positive endosome compartment and (ii) sorting into a Rab7-positive late endosome (LE), followed by ESCRT complex-mediated fusion into multivesicular endosome (MVE). The role of γ-secretase in EE and the release of N-ICD is less clear. From MVE, NOTCH proteins can be ectopically activated or MVE can fuse with the adaptors HOPS and AP-3 and, finally, with lysosomes, enabling NOTCH degradation. CCV: clathrin-coated vesicles; EE: early endosome; LE: late endosome; MVE: multi-vesicular endosome; N-FL: NOTCH full length; N-ICD: NOTCH intracellular domain.

Initial endocytosis studies focused on the role of ligand internalization. Loss-of-function mutations of *Shibire*, the *Drosophila* dynamin homolog [49], interfere with the ability of ligands to interact with NOTCH-presenting cells. However, endocytosis is not only reserved for ligands, but also for NOTCH receptors [50]. The trafficking of NOTCH receptors across cellular compartments is a limiting step in signal transmission. Briefly, NOTCH proteins travel through the ER/Golgi, from the cell surface to the nucleus and, eventually, follow degradation or recycling routes [51] (Figure 2). To do so, different E3-ubiquitin ligases, such as Deltex, Nedd4, and suppressor of Deltex/Itch (Su(Dx)/Itch for *Drosophila*/mammals), catalyze the monoubiquitination of NOTCH proteins [52] to trigger their internalization by early endosomes (EE) [51] in a clathrin-dependent manner [53,54]. NOTCH is subsequently sorted to other endocytic compartments, including recycling endosomes, multivesicular bodies (MVB)/late endosomes (LE), and lysosomes [51]. The removal of N-FL proteins is a key step at the EE level, wherein clathrin-endocytosed transmembrane proteins enter a GTPase Rab5 (Ras-related in brain)-positive compartment and are either directed towards the down-regulatory route or recycled back to the plasma membrane [51,55] by the action of Rab4 and Rab11 [55]. The selection between these two pathways may depend on covalent modifications or protein-binding partners. The non-activated NOTCH monoubiquitinated proteins are marked for degradation by the E3-ligases [56] that mature into intraluminal vesicles (IVs) and are degraded by an endosome-sorting complex required for transport (ESCRT). ESCRT consists of four (0–III) complexes that deliver ubiquitinated proteins into multivesicular endosomes (MVEs) [57]. MVEs fuse with lysosomes containing hydrolytic enzymes [58]. The ubiquitination of NOTCH residues is unrestricted to N-FL proteins. The regulation of N-ICD stability depends on the phosphorylation of the PEST domain. Phospho-PEST recruits the SCF/Sel10/FBXW7 E3-ubiquitin ligase complex to control N-ICD ubiquitination, turnover, and degradation by the proteasome (Figure 1). FBXW7 is an E3-ubiquitin ligase that targets, among others, N-ICD, MYC, and cyclin E [59], to mediate protein degradation. 

However, the endosomal structures are not exclusively produced for trafficking. Recent evidence suggests that some proteolytic events, including γ-secretase cleavages, may equally occur in EE and LE [60], which partly explain the ligand-independent mechanism of NOTCH activation. The model of ligand-independent receptor activation had been initially hypothesized in *Drosophila* [61], in which the loss of the E3-ubiquitin ligase Deltex led to a decrement of NOTCH internalization and, consequently, of its activation, suggesting that Deltex is implicated in the regulation of Notch endocytic trafficking. Deltex is an intracellular protein that binds to the cytoplasmic ankyrin repeats of NOTCH [62] and stabilizes it in the endocytic compartment to form a complex with adaptor proteins, such as AP-3 [63] and “homotypic fusion and vacuole protein sorting” (HOPS; [63]), that regulate NOTCH degradation. In fact, this complex shuffles NOTCH receptors to LE/MVB and is involved in endosomal trafficking toward the lysosome compartment, allowing the LE/lysosome fusion step [63,64] (Figure 2). During these steps in which the degradation drive is impaired, N-ICD is possibly released into the cytoplasm and, eventually, to the nucleus, through proteolytic cleavages in a ligand-independent way [65,66,67]. Secondary studies have confirmed that the loss of function of traffic-associated protein Lgd in *Drosophila* results in the ectopic activation of Notch signaling in a ligand-independent manner [68]. As previously mentioned, γ-secretase cleavage may occur in the endosomal compartment. Besides, some authors have shown a more efficient activity of γ-secretase in the endosomes due to their lower pH environment [60,69]; on the other hand, different observations have challenged this hypothesis by reporting the effective activity of γ-secretase in neutral pH conditions [70,71] and showing that, despite N-FL mutations that impair monoubiquitination, a less stable form of N-ICD can still be produced [72]. Hence, whether or not endosomal trafficking is necessary for N-ICD release is still not clear, being still under discussion. Furthermore, it may differ depending on the cell context.

## 6. Notch Signaling in Cancer

Notch regulates several cellular networks [73,74,75,76] and cross-talks whose pathways originate in close-contacting cells. It is not surprising that Notch signaling changes may contribute to oncogenesis, tumor maintenance, and escape [77,78]. Moreover, additional studies point to a role of Notch in immune surveillance [78,79] and in the tumor microenvironment (TME) [80,81,82]. Depending on the cell context, *NOTCH* may act as an oncogene or tumor suppressor but, more importantly, the degree of its contribution to the disease depends on the presence of activating or suppressing genetic aberrancies [77]. This heterogeneity explains why Notch downstream targets scarcely overlap across liquid and solid tumors. Only a few genes, such as *HES4*, *HEY1*, *MYC*, *NOTCH3*, *NRARP*, and *TFRC*, are similarly regulated in mutationally activated *NOTCH1*-driven cancers, such as T-cell acute lymphoblastic leukemia (T-ALL), mantle cell lymphoma (MCL), and breast cancer [83]. Through a collaborative effort, instead we showed that γ-secretase inhibitors (GSIs) regulate a similar set of genes in T-ALL and chronic lymphocytic leukemia (CLL), suggesting common mechanisms of transcriptional regulation in lymphoid malignancies [84].

### 6.1. NOTCH as an Oncogene

The transforming contribution of aberrant Notch signaling was initially described in T-ALL, an aggressive hematological malignancy that accounts for 15–20% of ALL cases [85]. In 1991, Ellisen and colleagues described a rare chromosome translocation, the t(7;9)(q34;q34.4), that juxtaposes the 3’ region of N1-ICD to the 5′ region of the *TCRβ* gene. This translocation generates a NOTCH1 fusion protein with ligand-independent activity [86]. Subsequent cancer modeling studies showed that aberrant N-ICD might lead to tumor formation or progression [87,88]. For example, exogenous expression of a *NOTCH1* plasmid lacking the N-ECD and N-TM domains in rat kidney cells led to the establishment of tumors in mice, compared to cells expressing wild-type *NOTCH1* proteins [87]. Similarly, transgenic mice overexpressing the *NOTCH1* homolog, *TAN1*, developed T-cell leukemia in 50% of the cases [88], supporting the notion that *NOTCH1* is a key oncogene in some cellular contexts. 

Gain-of-function *NOTCH1* mutations are sequenced in 50–70% of T-ALL cases [89]. These mutations alter NOTCH1 stability, causing differences in the extent of its ability to activate the downstream pathway [90]. The majority of aberrancies are class-1 mutations consisting of single amino acid substitutions, insertions, or deletions spanning the residues 1574–1622 on exons 26 and 27, corresponding to the HD-N and HD-C-terminal domains [89]. These mutations destabilize NOTCH1 heterodimers in two ways. In class 1A mutants, N-TM and N-ECD separate spontaneously, exposing the S2 cleavage site on N-TM to the activity of ADAM metalloproteases [90]. In class 1B mutants, the HD domain exposure to the S2 site is slower and can occur spontaneously or in denaturing conditions [90]. In 2008, Sulis and colleagues reported a rare group of mutations that account for only 3% of T-ALLs; these mutations involve the juxtamembrane expansion domain (JME), which displaces the HD-LNR repeat complex away from the cell surface to increase ligand-independent or ligand-hypersensitive NOTCH1 activation [91]. Class 2 HD domain mutations include in-frame insertions of 14 residues in exon 27, adjacent to the S2 cleavage site. These mutations reduce the protective effect of the HD-LNR complex on the S2 site, exposing the site to the activity of metalloproteases [89,90]. The second hot spot for the *NOTCH* mutations occurs in the PEST domain, accounting for 15–20% of T-ALLs. As previously mentioned, the PEST domain contributes to the stability and degradation of N-ICD through the ubiquitin-proteasome pathway, acting as a major route of self-limitation of the Notch pathway. Frameshift or non-sense substitutions in exon 34 alter the ubiquitin ligase FBXW7 binding site, leading to increased levels of N1-ICD and subsequent constitutive Notch activation [89,92]. Similarly, mutations involving the arginine sites on FBXW7 may lead to prolonged N1-ICD signaling in the nucleus [93,94]. Mutations affecting the Notch degradation pathway are weaker than class 1 and 2 mutations [89,90,95]. However, in 25% of the mutant cases, HD and PEST mutations may co-occur, enhancing the oncogenic activation of this pathway [89]. While *NOTCH1* mutations represent disease-causing events in T-ALL, their role as biomarkers has been debated [96,97,98,99]. Most pieces of evidence indicate that event-free survival (EFS) has positive predictive value in both pediatric [96,97] and adult T-ALL [99].

Under physiological conditions, Notch activation is required to commit common lymphoid precursors toward T-cell differentiation at the expense of early B-cell development by inductive inhibition [100]. However, fine-tuned control of Notch signaling and the lack of it dictate B-cell maturation and differentiation [100]. For example, the expression and activation by Dll1 ligand of NOTCH2 are critical for splenic marginal B-cell development [101]. Consistently, *NOTCH2* haploinsufficiency results in a severe reduction of splenic marginal zone B cells in Notch2^+/f^, CD19-Cre (Notch2^+/−^) mice models [101], while gain-of-function *NOTCH2* mutations occur in 10–25% of splenic marginal zone lymphomas (SMZLs) [102,103]. It may be possible that the acquisition of *NOTCH* mutations in B-cell malignancies is a late event in carcinogenesis that occurs in mature B-cells, conferring them a more aggressive phenotype [104]. From 5% to 15% of clinical cases of CLL and MCL present a 2 bp frameshift deletion (P2514 or 2515fs) involving exon 34 on the PEST domain [105,106,107,108]. In contrast to PEST deletions observed in T-ALL, PEST mutations in CLL and MCL span a larger region [89,109] and are associated with a poor prognosis, compared to the outcome in wild-type patients. In a series of 309 newly diagnosed CLL cases, the presence of a *NOTCH1* mutation conferred a >3-fold increased risk of death and shorter overall survival (OS) (*p* < 0.001). These patients presented with aggressive clinical features, an increased risk of Richter’s transformation, resistance to therapy, and a mutually exclusive correlation with *TP53* mutations [110]. Similarly, PEST mutations in diffuse large B-cell lymphoma (DLBCL) [107,111] are associated with a poor prognosis [112]. However, it has yet to be clarified how a single PEST mutation can activate Notch signaling. One hypothesis is that the interaction of malignant B-cells with the TME or with infiltrating macrophages leads to a cascade of events [113,114], culminating in the transactivation of Notch signaling. This may contribute to the development of mature B-cell malignancies, such as CLL and MCL [115,116]. Consistently, Dll4 triggers NOTCH1 expression in the PEST-deleted MCL cell line Mino, but not in the wild-type cell line Jeko-1 [117], and it rescues T-ALL or colorectal cancer cells from dormancy, thus activating NOTCH3 [118]. Collectively, these data suggest that, at least in some B-cell malignancies, *NOTCH* mutations cooperate with canonical signals and have obvious implications in cancer therapeutics.

The discovery of the oncogenic role of *NOTCH* in T-ALL anticipated the identification of *NOTCH* aberrations in solid tumors, such as lung adenocarcinoma [119], medulloblastoma [120], melanoma [121], colon cancer [122,123], and more frequently, in breast cancer [35,124]. Recent massive sequencing efforts have demonstrated that chromosome translocations are responsible for generating N1-ICD or N2-ICD peptides in 10% of triple-negative breast cancers [125] and oncogenic mutations occurring in *NOTCH4* genes [126,127]. Additionally, the expression of NOTCH1 and Jag1 correlate with poor overall survival [128].

Ultimately, the activation of Notch signaling can follow a non-canonical pathway in response to a functional relationship with other signaling pathways, such as the Ras, Wnt, or hypoxia-dependent pathways [129,130,131]. While these effects are less clear, they might confirm that altered Notch signaling is among the most frequent aberrantly activated pathways in human cancers.

### 6.2. Notch as a Tumor Suppressor

While *NOTCH* acts as an oncogene in the majority of cancers, its tumor suppressor role is firmly established in other cancers. Loss-of-function *NOTCH* mutations are frequently detected in epithelial-derived tumor histotypes, such as skin squamous cell carcinoma (SCC) [132,133], head and neck [134,135], esophageal [136], and bladder carcinoma [137]. Here, frameshift or non-sense mutations are predominant and lead to the generation of truncated peptides that are unable to communicate with the cognate ligands [133,134,135]. In SCC, *NOTCH1* mutations exceed those in *NOTCH2* and *NOTCH3* [138,139]. Furthermore, in SCC *NOTCH1* conditional knockout murine models, epidermal and corneal hyperplasia preceded cutaneous skin carcinoma [140], suggesting that *NOTCH1* deletion causes an aberrant proliferation of cells at the basal epidermal layer [141,142]. It is not surprising that GSIs increase the risk of skin cancer, as noted in murine models [140,143,144] or clinical trials using semagacestat [145]. On the contrary, the activation of Notch signaling in the skin, along with the Wnt and Sonic-hedgehog pathways [140], control differentiation and cell-cycle arrest through cross-talk with p21, interferon regulatory factor (IRF6), and the dual specificity phosphatases (DUSP) [137,142,146]. In this disease model, Notch controls tumor inflammation and progression [82,147] by providing a permissive microenvironment [82] in a non-cell-autonomous way. For example, *NOTCH1* deletion in keratinocytes results in the loss of skin barrier integrity, with the consequent increase of thymic stromal lymphopoietin (TSLP) expression by suprabasal keratinocytes that exhibit a potent proliferative effect on B-lymphopoiesis [82,148]. Furthermore, in a murine model of skin carcinogenesis, the tumor developing role of the loss of Notch signaling correlates with high levels of Wnt ligands and the subsequent increase in β-catenin signaling. Loss-of-function mutations in *NOTCH* lead to an impaired balance of tumor-protective/tumor-promoting inflammation by reducing the proinflammatory cytokine TSLP. TSLP allows the accumulation of CD11b^+^Gr1^+^ myeloid cells, which in turn, promote tumor growth by secreting Wnt ligands [149].

Among the non-SCC cancers, the tumor suppressor role of Notch signaling was described in hepatocellular carcinoma (HCC) [150] and glioma tumors [151]. In an *Rb*, *p107*, and *p130* triple-knockout (TKO) HCC model, transcriptional enrichment analysis of HCC cells showed the activation of pathways associated with Notch1-4 signaling and genes that activate canonical oncogenic signals, such as *Wnt*, *p38*, *Ras*, and *MAPK* [150]. Consistently, Notch inhibition with GSI DAPT accelerates tumor progression, while the overexpression of N1-ICD in cell lines derived from TKO tumors protects from tumor growth [150]. These observations indirectly confirm clinical HCC data, wherein the expression of the Notch target gene *HES1* correlates with a median OS of 43.7, months compared with 9.3 months, for those with low *HES1* expression [150,152]. However, two recent studies challenged these results and suggested the opposite role for Notch signaling in HCC progression [153,154]. Similar to that in the TKO HCC model, in a del*TP53* forebrain glioma tumor mouse, *NOTCH1*, *NOTCH2*, or *RBJP* knockout accelerates tumor progression, in part, by the downregulation of *HES5* [155,156]. Interestingly *NOTCH1-4* loss-of-function mutations have been identified in 10–31% and 7% of grade II and III glioma tumors, respectively [151,157,158].

In other cancer subtypes, such as pancreatic ductal adenocarcinoma (PDAC) or myeloid leukemia, the role of NOTCH is still not completely determined [95,109,138]. Genetic loss of *NCSTN* and the chemical inhibition of the γ-secretase complex in murine bone marrow or cord blood cells induce a hematopoietic phenotype similar to that of chronic myelomonocytic leukemia (CMML), with the aberrant accumulation of granulocyte/monocyte progenitors and extramedullary hematopoiesis [159]. Transcriptome analysis shows that NOTCH regulates a myelomonocytic gene signature through the downregulation of transcription factors *PU.1* and *C/EBPα* by *HES1* in early multipotential progenitor cells. Moreover, landscape studies have identified somatic heterozygous mutations in Notch pathway genes, such as *NCSTN*, *APH1*, *MAML1*, and *NOTCH2*, in 12% of CMML patients [159]. In acute myeloid leukemia (AML), the role of NOTCH is less clear. Most pieces of evidence suggest that Notch negatively regulates disease progression [160,161] through a cross-talk with the tumor microenvironment. On the other hand, an overactivated β-catenin pathway, due to a gain-of-function mutation, leads to Jag1 expression in mouse osteoblasts, inducing malignant changes through a NOTCH cell-autonomous effect in hematological myeloid progenitors; the genetic or pharmacological inhibition of NOTCH reduces the development of myeloid malignancies, supporting the pathogenic role of Notch pathway in this disease [162].

## 7. Targeting Notch Trafficking

While Notch signaling is regulated by enzymatic processes that are involved in small molecule inhibition, multiple roadblocks have been encountered in the translation of these drugs to a clinical setting; therefore, this has not been accomplished yet. Several studies in the literature have described these molecules [163,164,165] and strategies. Here, for simplicity, we focus on the approaches altering Notch trafficking (Table 1).

As described above, a furin-like protease (S1) processes an immature NOTCH form, in the ER/Golgi compartment, into a non-covalent heterodimer, that is subsequently expressed on the surface of cells [36] (Figure 1). In 1999, Goran Periz and Mark E. Fortini described that this process may be disrupted in the presence of a defective Ca^2+^-ATPase function in a *Drosophila* model. The loss of function of the *Drosophila* SERCA homologous gene *Ca-P60A* disrupts the proper synthesis, folding, and trafficking of the NOTCH receptor in the ER/Golgi compartment. Consistently, in *Drosophila* S2 lines, the treatment with SERCA inhibitors, such as thapsigargin and cyclopiazonic acid (CPA), reduces the amount of NOTCH protein on the cell plasma membrane [172]. Subsequently, Roti and Stegmaier confirmed these observations in a mammalian model of *NOTCH1* mutated T-ALL by integrating *NOTCH* gain- and loss-of-function screening approaches to identify inhibitors of oncogenic *NOTCH1* signatures or enhancer of *NOTCH1* HD mutant *L1601P∆P* activity [170]. The SERCA inhibitors and ion flux modulators scored highly in the screens, suggesting the feasibility of alternative approaches to enzyme inhibitors to suppress oncogenic *NOTCH1*.

Thapsigargin, a well-known anticancer agent [173], is extracted from the umbelliferous Mediterranean plant *Thapsia garganica*. Thapsigargin blocks SERCA in a Ca^2+^ free state through an irreversible lipophilic interaction in a so-called “dead-end” inactive state with low Ca^2+^ affinity [174,175]. SERCA inhibition impairs the trafficking of mutated NOTCH1 receptors from the ER to the cell surface, leading to the accumulation of aberrant N1-FL in the ER/Golgi compartment and the subsequent reduction of N1-TM, N1-ICD, and NOTCH1 target genes. NOTCH1 suppression causes cell cycle arrest and inhibition of leukemia growth in vitro in T-ALL xenografts and in a *Drosophila* intestinal stem cell model [170]. Importantly, thapsigargin preferentially targets mutated NOTCH1 proteins, as shown by its antileukemic effect and the lack of gastrointestinal toxicity in a human T-ALL xenograft murine model treated with 0.4 mg/kg thapsigargin, in sharp contrast with previous experiences with GSI [176]. However, translating the effects of thapsigargin into a clinical setting is limited by the generic effects on the SERCA pump, particularly on the cardiac isoform SERCA2a [177].

To overcome the limitations associated with the non-cell-specific effects of thapsigargin, Denmeade’s group developed a pro-drug approach to deliver thapsigargin only to cancer cells [178,179,180]. Following this enzymatic-based approach, Roti and colleagues leveraged the dependency of ALL on folic acid (FA) metabolism [181]. They first demonstrated that folate receptor 2 is aberrantly expressed in T-ALL, and then, they used it as an endocytic carrier for conjugated probes. Next, they showed that the conjugable alcohol derivative of thapsigargin, 8-*O*-debutanoyl-thapsigargin, inhibits NOTCH1 in a way that is similar to the effect of thapsigargin observed in T-ALL models. Notably, 8-*O*-debutanoyl-thapsigargin preserved a selective activity against mutant over *NOTCH1* wild-type T-ALL. Subsequently, they connected the carboxylate of folic acid to the C8-alcohol of 8-*O*-debutanoyl-thapsigargin via a cleavable ester linkage to generate JQ-FT. JQ-FT was 150 times more tolerable in mice than unconjugated thapsigargin and exerted an anti-leukemic effect in a preclinical *NOTCH1* mutated T-ALL in vivo model [181]. This approach sets the ground for the development of inhibitors with dual selectivity: cancer over normal cells and *NOTCH1* mutated over wild-type targets.

A different strategy that can be used to overcome the thapsigargin limitations due to cardiac effects is to search for new small molecules that share a therapeutic index similar to that of thapsigargin, but which have a safer toxicity profile. Beyond the few SERCA inhibitors with an unclear activity against Notch trafficking, such as curcumin [182,183,184] and CXL017 [185,186], the tricyclic clerodane diterpene Casearin J (CJ) was extensively explored as a NOTCH1 inhibitor in T-ALLs [187,188]. CJ affects Notch trafficking, reducing the cell surface expression of N1-TM and preventing the formation of cleaved N1-ICD modules, which results in the transcriptional suppression of Notch1 target genes, such as *MYC* and *HES1*. De Ford and colleagues also showed that CJ was more active against HD-mutated T-ALL cells than against a cell line carrying a *NOTCH1* juxtamembrane mutation, such as Jurkat [188]. Although this does not exclude the role of CJ on *NOTCH1* wild-type cells, it confirms that T-ALL is sensitive to Ca^2+^-ATPase suppression, further supporting the need to explore SERCA inhibitors with binding sites different from that of thapsigargin. Toward this goal, we completed an analysis of a small molecule screening of 191,000 compounds active on P-type ATPases; (4-[2-[2-[3-propyl-6-(trifluoromethoxy)-1H-indol-2-yl]-1-piperidyl]ethyl]morpholine) dihydrochloride inhibitor, CAD204520, showed favorable pharmacokinetics and dynamics profiles [189]. Both in preclinical and in vivo models, CAD204520 was well-tolerated without causing overt cardiac toxicity, while suppressing T-ALL leukemia growth. As CAD204520 binds to SERCA in residues different from the one occupied by thapsigargin, we may argue that the SERCA binding site influences the Ca^2+^ fluxes. This hypothesis is in line with the recent work of Sehgal and colleagues, which showed that SERCA inhibition may be achieved without triggering measurable changes in Ca^2+^ levels [190].

The original GE-HTS screen also led to the identification of the FDA-approved ion flux Ca^2+^ modulator, bepridil, a class IV antiarrhythmic drug [191] with limited toxic effects. This observation was leveraged to challenge a repurposing effort in *NOTCH1* mutated cancers. Bepridil showed preclinical activity in T-ALL [192] and CLL [84]. Similar to previous data on thapsigargin, bepridil impaired the trafficking of NOTCH1 proteins, resulting in the suppression of N-TM peptides and, consequently, the N1-ICD fragments in CLL. Interestingly, bepridil suppresses NOTCH1 activation in both cell-autonomous and non-autonomous conditions [193]. Consistently, ex vivo and in vivo studies confirmed that bepridil reduces CD45^+^CD19^+^CD5^+^ leukemic cells in the spleen and lymph nodes of mice. Interestingly, the anti-leukemic effects of bepridil were independent of the *IGHV* mutational status, *SF3B1*, *TP53* deletion, ZAP70 expression, and chromosomal abnormalities, such as 17p, 11q deletions, or 13q trisomy. Bepridil-treated mice showed no differences in body weight and no signs of gastrointestinal toxicities, further suggesting a preferential activity against NOTCH1 over NOTCH2. The number of goblet cells in the gut epithelium was preserved according to the PAS (Periodic acid–Schiff) staining distribution, suggesting a lack of NOTCH2 on-target effect [84].

A recent study by Fortini and colleagues showed that Notch trafficking can be impaired through the disruption of zinc (Zn^2+^) homeostasis in a *Drosophila* model; in a mutagenesis screen targeting genes controlling Notch trafficking, they demonstrated that loss-of-function mutations in the *Catsup* gene, a *Drosophila* orthologue of mammalian zinc transporter *ZIP7*, induced the accumulation of NOTCH protein in the ER/Golgi, and consequently impaired the signaling downstream to S1 cleavage. They also showed that the alteration of Zn^2+^ homeostasis led to increased ER stress levels and the activation of the unfolded protein response (UPR) [194]. Based on these premises, Nolin and colleagues completed a large, small molecule screening to identify ZIP7 modulators [171]. The authors showed that NVS-ZP7-1 and its derivative, NVS-ZP7-3, target the Notch pathway, as shown by the suppression of the Notch target genes *Deltex E3 Ubiquitin Ligase 1 (DTX1*) and *NOTCH3* by a mechanism resembling that of one of the SERCA inhibitors. Interestingly, they demonstrated an enhanced activity against the TALL-1 cell line that carries a *NOTCH3* activating mutation, suggesting that the disruption of ion homeostasis may cause a unique therapeutic index in wild-type vs. mutant. In contrast to our most recent work [189], the NVS-ZP7 small-molecule series trigger the activation of UPR. This stress effect, mediated by an accumulation of Zn^2+^ [171] in the ER, is similar to that caused by thapsigargin, urging further studies to assess the potential toxicities of this approach.

Post-translational *O*-glycans modification events occurring in the EGF domains are emerging targets for therapeutic intervention in NOTCH-dependent diseases. POFUT1 modulators represent the most successful example. As mentioned above, POFUT1 is responsible for the transfer of *O*-fucose to EGF repeats with an *O*-fucose consensus sequence. *O*-fucose modifications subsequently remain substrates for GlcNAc addition by enzymes of the Fringe family. Schneider and colleagues generated a series of analogs of GDP-fucose derivatives and tested their inhibitory effect on Notch signaling in transgenic Zebrafish Tg(Tp1bglob:eGFP)^um14^ embryos expressing a *NOTCH* reporter transgene (GFP under the control of elements responsive to N-ICD) [168]. In this series, compounds **3**, **4**, and **6** with C-6 ethynyl, ethenyl, and OH substituents, respectively, showed the greatest Notch inhibitory activity. The corresponding peracetylated fucose analogs (compounds **10** and **11** corresponding to compounds **3** and **4**) used for in vitro mammalian studies were transferred by POFUT1 onto NOTCH EGF repeats, replacing natural fucose on the polypeptides and suggesting a mechanism of Notch signaling disruption, rather than an inhibitory effect on the activity of fucosyltransferase or Fringe N-acetylglucosaminyltransferase. Compounds **10** and **11** abolished Notch signaling in a Dll1-4-Notch1 or Dll4-Notch2 reporter assay by disrupting the ligand-receptor binding but, intriguingly, caused no significant effects on Jag1-induced Notch signaling. Moreover, mutations of *O*-fucose residues on T311 in EGF8 and T466 in EGF12 rescued Notch activation in the aforesaid experimental model, suggesting that the presence of the fucose analogs interferes with NOTCH binding to Dll ligands. Collectively, these data pioneered a novel strategy to suppress Notch signaling by altering NOTCH glycosylation and present the first in class Delta-specific inhibitors of NOTCH1.

## 8. Targeting the Endosomal Compartment

Targeting endosomal trafficking appears as an emerging approach in tumors in which NOTCH is hyper expressed as a consequence of mechanisms other than mutations [195]. For example, in glioblastoma, components of the plasminogen activator (PA) system, such as urokinase-type plasminogen activator (uPA) and its receptor (uPAR), play a critical role in tumor progression and metastasis [196], regulating the proteolytic degradation of the extracellular matrix (ECM) [197], promoting tumor cell invasion, migration, and homing to distant organs [198]. Single (uPA, uPAR) or simultaneous (U2) genetic suppression of this multifunctional system reduces glioma cell invasion by inhibiting NOTCH1 cleavage and mRNA expression, as shown in commercially available (U251 MG) and xenograft-derived glioblastoma cell lines (5310 and 4910). Sequentially, *NOTCH1* downregulation reduces uPA expression, causing an anti-cancer positive feedback loop. Transcriptional suppression of uPA/uPAR accrues NOTCH1 accumulation in lysosomes, as shown in co-localization studies, suggesting that the disruption of the uPA/uPAR axis may, in turn, attenuate NOTCH1 receptor cleavage, signaling, and endosomal trafficking [199].

Another approach is by acting on the post-translational modifications required by NOTCH for trafficking or by acting on the endosomes that transport these receptors [65,200,201]. Histone deacetylases (HDACs), for example, control endosomal trafficking [202,203] by deacetylating histone or non-histone proteins, such as α-tubulin or Hsp-90 [204]. Consistently, trichostatin A, a pan HDAC inhibitor, reduces the level of N3-FL, N3-TM, and N3-ICD in two different human T-ALL cell lines and in different patient-derived xenografts (PDX), causing the transcriptional suppression of NOTCH3 target genes, such as pre-T cell receptor α (*pTα*), complement receptor (*CR*) *2* and *DTX1* [167]. Furthermore, NOTCH3 is retained in the endolysosomes upon treatment, as shown in immunofluorescence co-localization studies. Interestingly, genetic and enzymatic silencing of HDAC6 recapitulated these effects, suggesting that the selective suppression of HDAC6 may represent an alternative therapeutic approach in Notch3-dependent malignancies, such as T-ALL and glioma [167,205]. Collectively, these results support the development of selective approaches to disrupt the vesicular trafficking of NOTCH receptors.

## 9. Improving the GS Complex Inhibition Strategy

Several works have reviewed the role of γ-secretase in *NOTCH* mutated cancers [163,165,206]. However, few of them suggested the role of PSEN1 and PSEN2 in controlling γ-secretase cleavages in lysosomes, endosomes [60,72,207], or in the ER [208], suggesting that targeting PSEN complexes may lead to altered NOTCH, independently of the role of these proteins on S3 cleavage. Habets and colleagues showed that PSEN1 expression was higher than that of PSEN2 in T-ALL cell lines and patient samples, confirming previous evidence [209,210]. *Psen1*-knockout murine cells lack NOTCH expression, while *Psen2*-deficient cells maintain an adequate level of Notch signaling. These results support the hypothesis that PSEN1 selective inhibition may possess clinical activity in T-ALL. Treatment with the PSEN1 inhibitor MRK-560 decreases the leukemia burden and increases the overall survival in T-ALL patient-derived xenografts in vivo, without causing gastrointestinal toxicity or decreasing the normal T-cell development [169]. The lack of side effects may be explained by the compensatory activation of PSEN2 in the gastrointestinal tract and in developing T-cells [169]. These data also suggest that similar to SERCA inhibition, PSEN1 isoform-specific disruption preferentially affects mutated proteins over wild-type ones, overcoming the limitations associated with the first generation of GSIs [211,212].

A commonly used strategy to rescue GSI from their clinical outcome is the development of drug–drug combinations using anti-cancer [213] or anti-inflammatory agents [214]. The association of two potential anti-NOTCH agents has more rarely been attempted, given the risk of increased toxicity. Nevertheless, in a recent work, chloroquine (CQ), an autophagy inhibitor with known anti-cancer activity [166,215], enhanced the effect of GSI in *PTEN* wild-type T-ALL cell lines [166]. CQ reduces N-TM expression by altering the endosome-recycling of the protein. The few NOTCH proteins on the surface of cells remain as substrates for GSI inhibition. Overall, the data support the ongoing development of selective GSIs or alternative combination approaches for *NOTCH1* mutant T-ALL.

## 10. Conclusions

Given the high frequency of activating *NOTCH1* mutations in cancers, especially in T-ALL, the inhibition of the γ-secretase complex has represented a rational target to hijack the ligand-independent release of N-ICD for many years. Initial efforts attempted to repurpose GSIs, originally developed for Alzheimer’s disease [212,216], in relapsed/refractory T-ALL. However, only a few experiments reported therapeutic success [217,218,219]; for example, GSI 906024 developed by Bristol-Myers Squibb [220]. The CA216002 trial demonstrated the safety and tolerability of BMS-906024 administered weekly (4–6 mg) to 25 pediatric patients with T-ALL or T-cell lymphoblastic lymphoma [219]. Half of the patients reported, at least, a 50% reduction in bone marrow blasts, including a complete and a partial response. Despite these few encouraging results, the majority of drugs have not successfully passed clinical trials owing to toxicity secondary to non-specific proteolysis and a lack of therapeutic window across receptor isoforms (NOTCH1-4), and the alleles status (wild-type vs. mutated) [221]. Hence, given the clinical failure of first-generation GSIs [212], it is unlikely that such drugs will be further pursued as single agents. This is not the case for the selective PSEN1 inhibitor MRK-560, that demonstrated inhibition of mutant NOTCH1 processing and a decrease in the leukemia burden without causing gastrointestinal toxicity in T-ALL PDX in vivo. Similarly, in an orthotopic model of breast cancer treated with the monoclonal antibodies 10C11 and 2H6 targeting NCSTN, no histological evidence of goblet cell hyperplasia was detected, while these effects were evident in a GSI-treated arm [222]. Unfortunately, the therapeutic index of emerging NCSTN small molecule inhibitors, such as cowanin, for wild-type vs. mutant has not been tested yet [223]. Nevertheless, these encouraging results suggest that the selective disruption of members of the γ-secretase complex may preferentially affect mutated proteins, overcoming the innate limitations associated with pan γ-secretase inhibition.

Appropriate NOTCH subcellular localization is crucial and provides the time and space context for its activation. The succession of “S” cleavages is tightly regulated and, consequently, the subcellular mislocalization of NOTCH proteins might represent an attractive strategy for therapeutic interventions. We and others have been pioneers in the research of this strategy, identifying SERCA as a liable regulator of Notch signaling [170,172]. A recent study demonstrated that the disruption of *Drosophila* SERCA and Orai, a plasma membrane Ca^2+^ channel that mediates store-operated calcium entry (SOCE), produces three distinct outcomes in ER Ca^2+^ levels. While weak Ca^2+^ preferentially affects the Wnt Wingless (Wg) pathway, a more dramatic SERCA disruption affects the Hippo and Notch [224] signaling pathways, suggesting that Wnt-driven cancers may be the most sensitive to SERCA suppression. However, this level of granularity of ER Ca^2+^ control observed in experimental conditions may be difficult to achieve using thapsigargin, raising legitimate concerns regarding the effects caused by the abrogation of SERCA on the Hippo tumor suppressor pathway [224]. Nevertheless, we demonstrated that acute Ca^2+^ related toxicities or secondary consequences associated with the activation of UPR, associated with pan SERCA inhibition, might be overcome by the identification of new Ca^2+^ ATPase binding targets, to guide innovative drug development [189]. Moreover, in our experience, chronic exposure to SERCA inhibitors in several in vivo models did not cause a pro-tumorigenic effect [170,181]. Here, we can propose two hypotheses. First, our previous studies were too short in time to suppress Hippo signaling, and second, depending on the cell context, Hippo may have a tumor suppressor or an oncogenic role. Knockdown of the transducer yes-associated protein (YAP), a key modulator of Hippo signaling, decreased leukemia cell proliferation and enhanced cell apoptosis in T-ALL [225], suggesting that, in this context, Hippo might act as an oncogene. Although limited data have been reported from the clinical trial NCT01777594, no evidence of secondary tumors has been reported in patients treated with a thapsigargin derivative [179,180].

While these innovative approaches are yet to be translated into clinical practice, targeting Notch trafficking is within reach. For example, chloroquine, a well-known 4-aminoquinoline antimalarial drug, interferes with intracellular trafficking and processing of oncogenic NOTCH1 [166]. Due to the narrow therapeutic index, it is unlikely that this drug will affect highly proliferating cells as a single drug, but it would be promising to use it in combination with standard chemotherapy agents.

In conclusion, while GSI has dominated the arena of potential NOTCH inhibitors in the last two decades, new approaches targeting Notch trafficking are rapidly emerging. We expect that these strategies may be further explored for cancers in which oncogenic transformation requires sequential activations across different cellular compartments.

## Figures and Tables

**Table 1:** Active compounds that target Notch trafficking.

Chemical Structure	Class	Compound	Activity on Notch Trafficking	Ref.
** 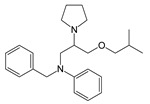 **	Antiarrhythmic class IV	Bepridil	Ion flux modulation	[84]
** 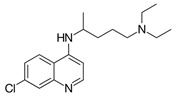 **	Antimalarial	Chloroquine	Endosomal trafficking impairment	[166]
** 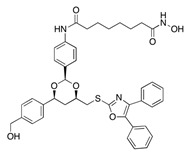 **	HDAC6 inhibitors	Tubacin	Endosomal trafficking impairment	[167]
** 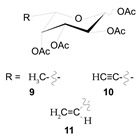 **	POFUT1 analogs	Compounds 9, 10, and 11	EGF-fucosylation inhibition	[168]
** 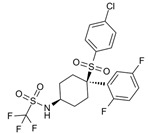 **	PSEN1 inhibitors	MRK-560	GS complex inhibition	[169]
** 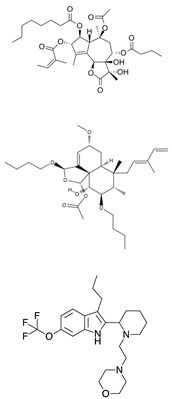 **	SERCA inhibitors	Thapsigargin Casearin J CAD204520	Ion flux modulation	[170]
** 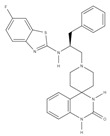 **	ZIP7 inhibitors	NVS-ZP7-4	Ion flux modulation	[171]
